# Organ Preservation in a Case of Retroperitoneal Ganglioneuroma: A Case Report and Review of Literature

**DOI:** 10.1155/2016/6597374

**Published:** 2016-09-07

**Authors:** Santosh Kumar, Shivanshu Singh, Abhishek Chandna

**Affiliations:** ^1^Department of Urology, Postgraduate Institute of Medical Education and Research (PGIMER), Chandigarh, India; ^2^Department of General Surgery, Postgraduate Institute of Medical Education and Research (PGIMER), Chandigarh, India

## Abstract

The retroperitoneum is a closed space harbouring vital organs including the great vessels, kidneys and adrenal glands, ureters, and the ascending and descending colon. Surgical management of retroperitoneal pathologies may need multiorgan resection in order to achieve complete surgical resection while preservation of surrounding organs should be attempted, especially in case of benign tumors. We present a case of 15-year-old girl with an 11 × 6 × 5 cm retroperitoneal ganglioneuroma displacing the right kidney, renal vein, and ureter and abutting the IVC which was excised in toto preserving the right kidney and ureter with careful dissection around the great vessels. We also attempt to review the various surgical options available while dealing with these benign retroperitoneal tumors which are often detected incidentally and usually surround important retroperitoneal organs and vessels.

## 1. Introduction

The retroperitoneal space harbours various vital structures including the great vessels, kidneys and adrenal glands, duodenum, ascending and descending colon, and the ureters. This calls for extreme caution and expertise while dealing with pathologies arising from this space. Management of tumors arising in the retroperitoneal space often requires multiorgan resection and reconstruction in order to achieve complete resection of the tumor [1]. Careful dissection and preservation of these structures is paramount, especially in case of benign retroperitoneal masses.

We present a case of a 15-year-old girl with a retroperitoneal mass displacing the right renal vein and ureter, compressing the inferior vena cava and infiltrating the right psoas muscle. The tumor measuring 11 × 6 × 5 cm was excised with careful dissection around the IVC and preservation of the right kidney, ureter, and right renal vein. Histopathology revealed retroperitoneal ganglioneuroma with no undifferentiated component.

## 2. Case Presentation

**Figure 1:**
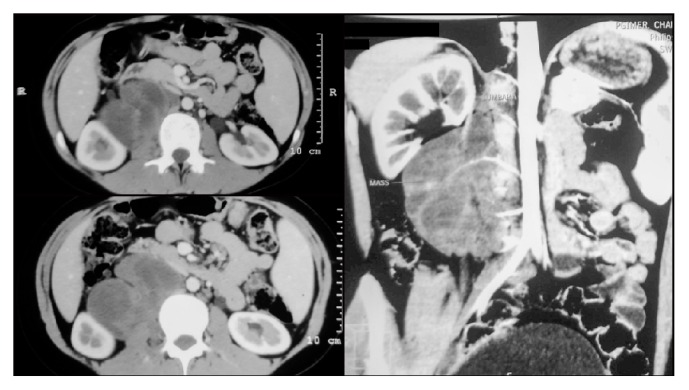
Axial and coronal sections on CECT abdomen and pelvis showing the extent of the tumor.

A 15-year-old female presented with complaints of pain in the right lower limb and back for 1 month. Ultrasound revealed a retroperitoneal mass adjacent to the lower pole of the right kidney. CT scan revealed a large heterogeneous lobulated mass with intralesional cystic areas of necrosis and heterogeneous postcontrast enhancement in the retroperitoneum measuring 11 × 5.5 × 6 cm in size on the right side at the level of mid and lower pole of the kidney. There was no evidence of calcification in the lesion. The mass had extraneous indentation of the inferomedial cortex of the right kidney. The mass displaced the right renal vein anteriorly and the right proximal ureter anterolaterally with compression of the inferior vena cava anteromedially. Medially, the mass was seen to be infiltrating the psoas muscle and abutting the right lateral cortical margin of the L4 vertebra. The right adrenal gland was seen separately and appeared normal (Figure 1). 3D reconstruction of the imaging was done to assess the relation of the mass with the surrounding structures.

Serum cortisol and serum and urine normetanephrines were normal, ruling out a functional tumor. The patient underwent USG-guided core needle biopsy which revealed retroperitoneal ganglioneuroma as presence of mature ganglion cells in a Schwannian stroma with no evidence of mitosis/necrosis.

The patient underwent exploratory laparotomy through anterior subcostal incision. The mass was found to be displacing the right kidney and proximal ureter laterally with anterior displacement of right renal vein. The right ureter was looped using a vascular sling and traced upwards to identify the right kidney and the right renal vein. The mass was carefully dissected from the surrounding structures using electrocautery and the base of the mass infiltrating the psoas muscle was fulgurated.

**Figure 2:**
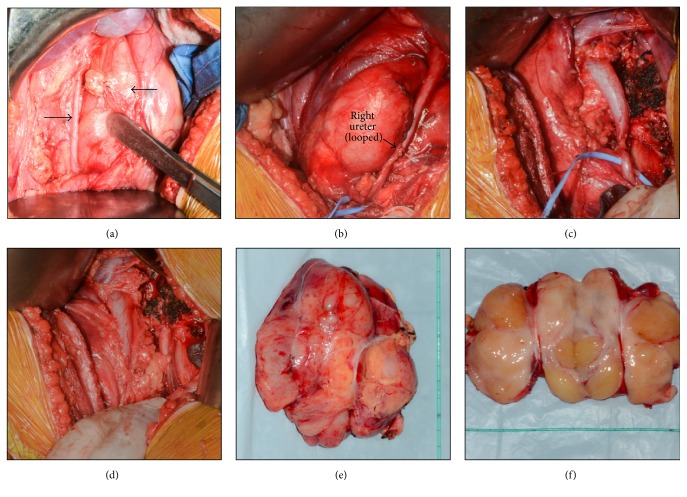
(a) The intraoperative findings: left arrow indicating the retroperitoneal mass; right arrow indicating the ureter. (b) The ureter looped and traced. ((c), (d)) Tumor excised and base fulgurated. (e) The resected tumor. (f) Cut section of the tumor.

The mass was encapsulated and measured 11 × 6 × 5 cm in size. It had a bosselated surface. The cut surface was homogeneous and soft to firm in consistency with no haemorrhage or necrosis (Figure 2).

The postoperative course of the patient was uneventful. The histopathology revealed a biphasic tumor with ganglion cells and Schwannian stroma. The ganglion cells were mature with eccentric nuclei, prominent nucleoli, and abundant eosinophilic cytoplasm. No undifferentiated component was seen in the tumor. The final histopathological diagnosis was retroperitoneal ganglioneuroma.

Postoperatively, the patient has been relieved of her symptoms, and in the two years of follow-up, there is no evidence of local or distant recurrence so far.

## 3. Discussion

Ganglioneuroma is a rare (one per million population [2]), differentiated, benign, slow-growing tumor that commonly arises from sympathetic ganglion cells and is composed of mature Schwann cells, ganglion cells, and nerve fibres [3]. Ganglioneuromas may arise anywhere along the paravertebral sympathetic plexus and occasionally from the adrenal medulla. Common sites of origin are retroperitoneal (32% to 52%), mediastinal (39% to 43%), or cervical (8% to 9%) region [4]. Although ganglioneuromas usually develop in childhood, they are often detected in adults because they grow slowly. Two-thirds of patients are under the age of 20 years and ganglioneuromas are rarely observed over the age of 60 years [5].

Patients with ganglioneuroma are usually asymptomatic or present with nonspecific symptoms. Symptoms occur as a result of mass effect and compression of surrounding organs with signs and symptoms predictable when tumor enlargement causes elevation of the diaphragm, interference with portal structures, distortion and subsequent disturbance in functions of the genitourinary or gastrointestinal tract, pressure on the spinal cord, peripheral nerves or nerve plexuses, and distortion and erosion of bony structure [1, 6–8]. Palpable abdominal mass as well as abdominal or back pain can be observed in patients with tumor located in the retroperitoneum as was observed in our patient [6]. Ganglioneuromas are generally hormonally inactive but they may produce catecholamines and other hormones. This is rarely found in mature ganglioneuromas. The patients with hormone producing tumors (catecholamines, vasoactive-intestinal peptides) may present with signs and symptoms of sympathetic overactivity like hypertension, diarrhoea, and flushing [3, 7, 9].

The imaging modality of choice is contrast enhanced CT scan and MR imaging. They help in identifying the relationship of the tumor with surrounding organs and vital structures. The surgical approach to the tumor is also decided on the basis of the imaging findings. On CT and MR images, ganglioneuromas usually present as an oval, well-defined mass in the adrenal gland or in extra-adrenal location. Ganglioneuromas characteristically have low attenuation on unenhanced CT scans [5]. Cai et al. [10] studied retroperitoneal ganglioneuromas in children and found a correlation between the attenuation of tumor on CT scan and the proportion of myxoid stroma to cellular components and collagen fibres. On MR imaging, ganglioneuromas show homogeneous low signal intensity on T1-weighted images and inhomogeneous high signal intensity on T2-weighted image [5, 10, 11]. Another imaging feature of ganglioneuromas is delayed enhancement to varying degrees on CT and MR images [10].

The definitive diagnosis is made on histopathology. In our case, an ultrasound guided biopsy was done prior to going in for surgical resection to confirm the diagnosis. The treatment for retroperitoneal ganglioneuromas is surgery and they bear excellent prognosis following complete surgical excision [12–14]. Their close relationship with surrounding vital organs and inherent propensity of these tumors to surround blood vessels makes surgical excision of these tumors challenging [14–16] and may act as a limiting factor in complete surgical resection [17]. Ganglioneuromas, being benign tumors, call for meticulous dissection and preservation of surrounding structures while operating on these tumors.

The surgical resection of these tumors can be undertaken via laparoscopic or open technique. Laparoscopic surgery is increasingly becoming common for retroperitoneal pathologies with increasing expertise and availability of advanced laparoscopic instruments and energy sources. In our case, open surgery was conducted owing to the close relation of the tumor with the IVC and displacement of the right renal vein and right ureter along with the large size of the tumor.

Alimoglu et al. [7] described the en bloc laparoscopic excision of a 50 mm retroperitoneal tumor with careful dissection from the celiac trunk, segment I of liver, pancreas, and overlying left gastric and hepatic artery. Zografos et al. [3] and Abraham et al. [18] have described laparoscopic excision of adrenal ganglioneuroma measuring 13 × 9.5 × 6 cm and 17 × 11 × 7.5 cm in independent case reports. These case reports suggest complete surgical excision of even large tumors is possible laparoscopically and is associated with shorter postoperative stay, fewer complications, and less bleeding.

Open surgery has often been preferred while operating on tumor in close proximity of or surrounding important blood vessels in the abdominal cavity where laparoscopy may face limitations. Vasiliadis et al. [14] reported an extra-adrenal ganglioneuroma involving the infrahepatic IVC, superior mesenteric artery, and celiac axis which was excised via an upper midline laparotomy. The tumor measured 7 × 4 × 3 cm and was approached after mobilizing the left hemiliver and the structures of the portal triad were driven away from the right margin of the tumor after encircling the hepatoduodenal ligament using a Penrose drain. Acín-Gándara et al. [12] reported the excision of a 15 cm retroperitoneal ganglioneuroma encompassing the celiac axis and superior mesenteric artery and extending from the right renal vein to the diaphragmatic hiatus using a midline laparotomy after mobilization of the pancreas and the liver.

Some authors have also described novel techniques in order to achieve complete resection without compromising the surgical clearance and also preserving surrounding structures. Wan et al. [16] described the technique of “fractionated resection” used for tumors surrounding important blood vessels. They used this technique for a retroperitoneal tumor 7.2 × 4.3 × 7.2 cm in size completely surrounding the celiac axis and the splenic, common hepatic and superior mesenteric arteries and closely associated with the abdominal aorta and the portal, splenic, superior mesenteric and left renal veins. Additionally, the mass invaded the pancreatic head and compressed the pancreatic body being inseparable from the pancreatic head. Bounded by the splenic and common hepatic arteries, the tumor was divided into upper and lower fractions and was incised along the course of these vessels exposing the celiac axis, and the left gastric artery was severed. Also bounded by the celiac axis and the superior mesenteric artery, the tumor mass was further divided into left and right fractions and incised along their course exposing the SMA and celiac axis to the abdominal aorta. The tumor fractions were resected separately in the following sequence: upper right, lower right, upper left, and lower left protecting the blood vessels. This was followed by pancreaticoduodenectomy for the mass was inseparable from the pancreatic head.

Oue et al. [19] have also described a total laparoscopic approach to retroperitoneal ganglioneuroma using the “hanging method” and a vessel sealing device. They described the case of an 11-year-old girl with 5.5 × 4.8 × 3 cm right retroperitoneal tumor behind the duodenum and ascending colon located anterior to the upper pole of the right kidney and extending along the anterior aspect of the IVC with no involvement of the renal vein or IVC. After medial mobilization of the duodenum and ascending colon, the tumor was carefully dissected free of the surrounding structures and the IVC running posterior to the lesion was identified and protected. In order to create a space behind the tumor, they stitched a 3-0 suture to the tumor and suspended it in the retroperitoneal space. The tumor was dissected from the IVC and haemostasis was achieved using a vessel sealing device (endoscopic LigaSure; Tyco Healthcare, Gosforth, UK) preventing bleeding the small vessels between the IVC and the tumor.

Major vascular resections and prosthetic replacements have also been performed for retroperitoneal tumors. Fueglistaler et al. [17] reviewed 8 patients who underwent resection of infrarenal aorta or infrarenal IVC (or both) as a part of oncologic procedure for retroperitoneal tumors with prosthetic replacement. One patient was an 11-year-old patient who underwent aortic tube replacement as part of the surgery for recurrent ganglioneuroma. The graft remained patent on follow-up and the patient had no local or distant recurrence during the period of follow-up of the study. This suggests that vascular resections with or without prosthetic implants may be considered for retroperitoneal ganglioneuromas but must be used as a last resort especially in case of recurrent tumors. But complete surgical resection should always be the objective during treatment of these patients with preservation of surrounding organs and vascular structures as far as possible.

Certain principles that may be kept in mind while operating on benign tumors in the retroperitoneum are as follows:

- Thorough knowledge of the retroperitoneal anatomy, along with careful and meticulous dissection, is an absolute must in order to avoid injury.
- CECT and 3D reconstruction may help the surgeon in identifying the relationship of the tumor with the surrounding structures and plan the approach to the tumor preoperatively.
- Vascular structures and ureter may be looped using slings; ureter can also be stented using DJ stents or ureteral catheters.
- Threshold is low for conversion to open surgery when laparoscopic approach was used in favour of avoiding injury to neurovascular structures.
- Use of energy sources such as harmonic scalpel may be preferred avoiding lateral thermal injury.
- Adherence to oncologic principles and complete surgical excision as residual tumor may lead to local recurrence.

## 4. Conclusion

Complete surgical resection of benign retroperitoneal tumors calls for meticulous and novel surgical techniques in order to preserve surrounding vital organs. With increasing expertise, this may be achieved laparoscopically as well but each case must be individualized on a case-to-case basis without compromising on oncological principles while dealing with these tumors.
